# Therapeutic Vaccines for Follicular Lymphoma: A Systematic Review

**DOI:** 10.3390/ph17030272

**Published:** 2024-02-21

**Authors:** Andrei Suponin, Pavel Zhelnov, Artem Potanin, Andrey Chekalov, Aleksandr Lomazov, Kseniia Vladimirova, Kirill Lepik, Albert Muslimov

**Affiliations:** 1 RM Gorbacheva Research Institute, Pavlov University, L’va Tolstogo 6-8, 197022 St. Petersburg, Russia; andrey.suponin2011@gmail.com (A.S.); artempotanin28@gmail.com (A.P.); a.m.chekalov@gmail.com (A.C.); alex.lomazov@icloud.com (A.L.); ksenia.vladimirova2002@gmail.com (K.V.); lepikkv@gmail.com (K.L.); 2 Independent Researcher, Mexico City 07780, Mexico; pavel@zheln.com; 3 Laboratory of Nano- and Microencapsulation of Biologically Active Substances, Peter the Great St. Petersburg Polytechnic University, Polytechnicheskaya 29, 195251 St. Petersburg, Russia; 4 Center for Molecular and Cell Technologies, Saint-Petersburg Chemical-Pharmaceutical University, Professora Popova 14, 197376 St. Petersburg, Russia

**Keywords:** non-Hodgkin indolent lymphoma, immunization, individual participant data reconstruction

## Abstract

(1) Background: We aimed to estimate the pooled effectiveness and safety of vaccination in follicular lymphoma (FL) and discuss implications for immunotherapy development. (2) Methods: We included randomized trials (RCTs) of therapeutic vaccines in patients with FL. Progression-free survival (PFS) was the primary outcome. We searched databases (PubMed, Embase, Scopus, Web of Science Core, medRxiv) and registries (PROSPERO, CENTRAL, ClinicalTrials.gov, EuCTR, WHO ICTRP) and conducted online, citation, and manual searches. We assessed risks of bias across outcomes using RoB 2.0 and across studies using ROB-ME and a contour-enhanced funnel plot. (3) Results: Three RCTs were included (813 patients, both previously treated and untreated). Patients with a complete or partial response after chemotherapy were randomized to either a patient-specific recombinant idiotype keyhole limpet hemocyanin (Id-KLH) vaccine plus granulocyte–macrophage colony-stimulating factor (GM-CSF) or placebo immunotherapy (KLH + GM-CSF). Meta-analyses showed that PFS was worse with the vaccine, but not significantly: hazard ratio, 1.09 (95% CI 0.91–1.30). The GRADE certainty of evidence was moderate. Adverse event data were mixed. (4) Conclusions: We are moderately certain that Id-KLH results in little to no difference in PFS in FL. (5) Funding: Russian Science Foundation grant #22-25-00516. (6) Registration: PROSPERO CRD42023457528.

## 1. Introduction

Follicular lymphoma (FL) is a tumor that originates from germinal center (GC) B-cells and is characterized by certain histological features: predominance of follicular morphology and preservation of GC architecture; immunophenotypic features, where FL B-cells express GC markers BCL-6+ and CD10+; and molecular features indicating ongoing class-switch recombination and somatic hypermutation. An important clinical characteristic of FL is its indolent course, with periods of relapse and remission, and remission periods gradually shortening with disease progression [1].

FL is the most common subtype of indolent lymphoma and the second most common subtype of all lymphomas. FL in the United States and Western Europe accounts for approximately 35% of all non-Hodgkin lymphomas (NHLs), and 70% of indolent lymphomas [2]. The incidence of FL in the US is approximately 3.18 cases per 100,000 per year, and in Europe the incidence is approximately 3.14 cases per 100,000 per year [3,4]. Moreover, the number of cases increases with age: in the age group of 60–70 years, the incidence rate is already 10 people per 100,000, and the median age at diagnosis is 65 years [3,5]. It is worth noting that the incidence of FL is slightly higher for relatives of people with FL [6]. 

FL cells are derived from normal germinal center B cells blocked at the differentiation stage. The molecular hallmark of FL is the t(14;18)(q32;q21) translocation, with the BCL2 gene placed under the control of the IGH locus, which leads to overexpression of the anti-apoptotic protein BCL2 [1]. However, only 70–90% of patients with FL have this mutation, and the t(14;18) translocation can also occur in B cells of healthy people and patients with other tumors [7]. Another important molecular characteristic of FL is the high frequency of mutations disrupting epigenetic regulation, with more than 90% of patients having mutations in one or more chromatin-modifying genes [1]. As an example, the following genes can be mentioned: KMT2D, CREBBP, EZH2, EP3000 [8].

FL is characterized by an indolent course. The most common symptom is asymptomatic lymphadenopathy, which may wax and wane for years. Extranodal lesions most often affect the bone marrow; other organs are affected much less frequently. Many patients have no symptoms, but some present with B symptoms [9]. FL can undergo histological transformation into diffuse large B cell lymphoma, which is characterized by a more aggressive course with the rapid progression of symptoms and a worse prognosis [10].

The management of FL depends on many factors, and there is still no consensus on the optimal choice of treatment options. In the early stages (Ann Arbor classification stage 1/2), local radiotherapy is preferred, to which rituximab, as an anti-CD20 antibody, may be added, and a watch-and-wait strategy or systemic therapy may also be considered. For newly diagnosed advanced FL (Ann Arbor classification stage 3/4), a watch-and-wait strategy may also be considered for asymptomatic patients [10,11]. The Groupe d’Etude des Lymphomes Folliculaires (GELF) criteria or the British National Lymphoma Investigation (BNLI) criteria can be used to decide whether to initiate therapy [12,13]. 

If the decision to start therapy is made, then the method of choice is chemoimmunotherapy. Adding rituximab to a chemotherapy regimen improves progression-free survival (PFS) and overall survival (OS) [14]. The most commonly used chemotherapy regimens are Rituximab + Cyclophosphamide, Doxorubicin, Vincristine, and Prednisone (R-CHOP); Bendamustine + Rituximab (BR); and Rituximab + Cyclophosphamide, Vincristine, and Prednisone (R-CVP). In addition, monotherapy with rituximab may be considered. Obinutuzumab, another anti-CD20 antibody, may be an alternative to rituximab [10]. The lenalidomide–rituximab combination has the same efficacy as immunochemotherapy [15].

For patients with relapsed and refractory FL, treatment will depend on the effectiveness of previous regimens, duration of response, and stage of relapse. To treat relapses, the treatment regimens described above can be used, as well as radioimmunotherapy—ibritumomab-tiuxetan, a phosphoinositide 3-kinase (PI3K) inhibitor idelalisib, and autologous/allogeneic hematopoietic stem cell transplantation (HSCT) [15]. A T-cell-engaging CD20 × CD3 bispecific monoclonal antibody mosunetuzumab could be named as a new promising therapeutic agent for relapsed and refractory FL management. A recent phase 2 study demonstrated a significant increase in complete response rate with Mosunetuzumab therapy (60.0% [95% CI, 49.1–70.2]) compared with the historical control complete response rate with copanlisib (PI3K inhibitor) of 14% [16]. 

However, at present, genetic engineering therapy plays an increasingly important role in the treatment of various diseases. FLs are no exception. At present, CAR-T and antitumor vaccines are promising methods of therapy.

CAR-T therapy is an extremely effective and relatively safe treatment for relapsed/refractory forms of FL. Currently, two anti-CD19-specific drugs are registered with the U.S. Food and Drug Administration (FDA)—axicabtagene ciloleucel (axi-cel) and tisagenlecleucel. Both drugs show high response rates, complete remission rates, 2-year OS (axi-cel), 1-year PFS (tisagen-lecleucel), and a low rate of predictable severe complications [17,18]. It is worth saying that, at present, there are no studies directly comparing CAR-T and other lines of therapy [19]. However, from the available indirect evidence, CAR-T has a higher complete remission rate than PI3K and EZH2 inhibitors, and better tolerability and lower mortality compared with allo-HSCT [19,20].

Antitumor vaccines are a type of active immunotherapy aimed at developing an immune response to certain tumor antigens. There are significant differences between cancer and traditional vaccines. Traditional vaccines aim to either prevent an infectious disease or, if it develops, to alleviate the disease. In addition, traditional vaccines only affect humoral immunity. At the same time, CD8+ cellular immunity mediated by cytotoxic T cells is crucial to ensure the destruction of malignant cells in the creation of anti-tumor vaccines [21]. Also, in the case of antitumor vaccines, it is difficult to find a suitable tumor-specific antigen against which an effective immune response can be generated. Most tumor-associated antigens are also expressed on normal human cells, and, normally, the immune cells that are able to recognize them are a target of immune tolerance mechanisms [22].

Depending on the form of the delivered antigen, vaccines can be divided into several types: peptide (proteins of cancer antigens), cellular (dendritic cells loaded with tumor antigens, lysed tumor cells), and those using DNA/RNA with encoded antigen sequences (plasmids, viral vectors, various RNA-based platforms) [23]. Antitumor vaccines can also be divided into autologous and allogeneic. Autologous vaccines include dendritic vaccines, and allogeneic vaccines include peptide vaccines, DNA vaccines, mRNA vaccines, and adenovirus vaccines [24,25].

Antitumor vaccines have long attracted attention from scientists. At present, there are many preclinical and clinical studies for different types of tumors, but only two therapeutic cancer vaccines (sipuleucel-T for prostate cancer [26] and talimogene laherparepvec (T-VEC) for melanoma [27]) were approved by the FDA and the European Medicines Agency (EMA) [28].

Vaccines directed against FL have been the subject of clinical studies for many years, but no systematic review has been completed on this topic until now. In 2011, Ossendorf et al. published a Cochrane protocol dedicated to idiotype vaccination for non-Hodgkin lymphoma, but it was withdrawn in 2016 due to a lack of progress. We have reviewed and gained insights from this protocol.

This work was inspired by the recent successes of mRNA vaccines. The synthetic mRNA format proved to be the fastest and safest, and highly effective, even at the low dose of a few micrograms per injection. Nucleic-acid-based vaccines have several advantages over other types of vaccines, such as stimulating a strong specific immune response, the ability to assist in antigen detection, their enabling the rapid screening of candidate vaccines, their common manufacturing platform, and their relatively low development costs. In addition, the problem of intracellular delivery of nucleic acids is likely to be overcome over time, and progress in this field has the potential to increase the effectiveness of such vaccines dramatically.

The aim of this study is to evaluate the effectiveness and safety of therapeutic vaccines in patients with FL, in order to form the basis for further work on the development of therapeutic mRNA vaccines for FL. In our review, we focus on the results of clinical trials of FL vaccines and discuss possible strategies for increasing their effectiveness in the future.

## 2. Materials and Methods

This systematic review was conducted using the Cochrane Handbook version 6.4 [29] and reported in accordance with PRISMA 2020 [30] and PRISMA-S [31]. The protocol was developed and prospectively registered in PROSPERO: https://www.crd.york.ac.uk/prospero/display_record.php?ID=CRD42023457528 (accessed on 10 January 2024).

### 2.1. Eligibility Criteria

#### 2.1.1. Types of Studies

We considered any randomized controlled trials (RCTs) with no restrictions on language or date of publication. Commentaries and editorials were ineligible for inclusion. Systematic reviews and study protocols were not included but were reviewed for eligible references.

#### 2.1.2. Types of Participants

Patients of any age, sex, and race with a histologically confirmed diagnosis of FL and any stage or grade of disease were eligible. We considered patients who had already undergone any type of therapy for FL, as well as patients who had not been treated for FL prior to trial enrollment. If patients with other types of lymphoma were recruited along with patients with FL in the study under consideration, we would include such studies whenever possible and select data relating only to patients with FL.

Pre-clinical studies, such as in vitro, in silico, or animal models, were ineligible.

#### 2.1.3. Types of Interventions

We considered therapeutic antitumor vaccines. Therapeutic antitumor vaccines were defined as interventions designed to induce and/or enhance a specific immune response against tumor-associated and/or tumor-specific antigens. The following types of vaccines were eligible [25]:

- Autologous therapeutic vaccines:
  ◦ Vaccination with inactivated autologous tumor cells and adjuvant;
  ◦ In situ vaccination;
  ◦ Autologous dendritic cell vaccines.
- Allogeneic therapeutic vaccines:
  ◦ Tumor-associated antigen-based vaccines;
  ◦ Neoantigen-based vaccines;
  ◦ Idiotype therapeutic vaccines.

#### 2.1.4. Types of Outcome Measures

We did not review outcomes at the report-selection stage.

### 2.2. Information Sources

We searched an extensive range of information sources, including bibliographic databases (Embase Classic and Embase through Ovid; Scopus; PubMed; several Web of Science databases, including the Core Collection), preprint servers (bioRxiv, medRxiv, and other preprint servers via the OSF Preprint Archive Search), and study registries (PROSPERO; Cochrane Central Register of Controlled Trials; ClinicalTrials.gov; EU Clinical Trials Register; other study registers via the WHO International Clinical Trials Registry Platform Search Portal). The choice of information sources was justified based on the Cochrane Handbook for Systematic Reviews of Interventions version 6.4 [29] and specific guidance for register searches [32]. Please refer to the search strategies report in the review repository for a full listing of information sources (see the Data Availability Statement).

Reference lists of the included reports were screened for additional potentially eligible records by A.S. On 22 January 2024, P.Z. conducted a Scopus-powered forward citation search based on the three main reports of the included studies, and A.S. scanned the results or potentially eligible reports.

As suggested by an anonymous peer reviewer, we supplemented electronic searches with manual searches of relevant journals and conference proceedings to enhance the completeness of the coverage. We reviewed the official websites and conference proceedings databases of major professional societies in hematological oncology, namely the American Society of Clinical Oncology (ASCO) and Journal of Clinical Oncology/ASCO Publications, American Society of Hematology (ASH) and Blood, and the European Hematology Association (EHA). Of note, all three of the included studies were reported in these journals [33,34,35].

We did not plan to contact study authors but did contact them eventually in an attempt to procure individual participant data for analyses (see Section 3.2). 

### 2.3. Search Strategy

We leveraged the search strategies developed by several Cochrane teams [36,37,38,39]. Please refer to the search strategies report in the review repository for the full search strategies used for each database and register (see the Data Availability Statement).

We supplemented the initial searches with a series of Google searches, as well as searches in the aforementioned trial registries, using the identified vaccine names (“BiovaxID”, “Specifid”, “MyVax”) to identify additional reports of the included trials.

### 2.4. Study Records

#### 2.4.1. Data Management

The review is hosted in an Open Science Framework repository [40]. P.Z. was responsible for managing the repository and all other data and software in the course of this review. Screening was conducted in Rayyan [41]. Data extraction was completed using sysrev.com, free edition.

#### 2.4.2. Selection Process

We deduplicated records automatically using the Thorough (Focused) Algorithm of Systematic Review Accelerator’s Deduplicator [42], then manually using in-built Rayyan deduplication features. The reported diagnostic sensitivity and specificity of an early version of this deduplication algorithm are 84% and 100%, respectively [43], suggesting that it “may mislabel non-duplicates but misses very few actual duplicates” [44]. After deduplication, we applied RobotSearch, a support vector machine (SVM) classifier trained for identifying RCTs. We used Taren Sanders’ fork of RobotSearch [45], which is up-to-date for current Python setups. In the original evaluation, RobotSearch performed with a promising area under a receiver operating characteristic curve (AUROC) of 0.987; 95% CI, 0.984–0.989 [46].

For pilot screening, we randomly selected 500 records from the deduplicated record set and uploaded them into Rayyan. We completed randomization of the pilot set using the RANDOM.ORG Integer Set Generator in Advanced Mode [47]. The rationale for choosing the pilot sample size was as follows: Based on the prevalence of “vaccine” (6.6%, or 581 hits in 8745), “[a] minimum of 423 subjects are required for this study of interobserver agreement” (CIBinary(kappa0 = 0.75, kappaL = 0.6, kappaU = 0.9, props = c(0.934, 0.066), raters = 2, alpha = 0.05)) (R package kappaSize version 1.2 [48]). Given that the actual inclusion rate might have been even lower, e.g., 6% (a minimum of 462 subjects), a sample size of 500 appeared appropriate.

At the end of the piloting procedure, we calculated Randolph’s free-marginal multirater kappa [49] to measure inter-rater agreement for the screeners (2 raters; 2 categories—Included or Excluded; 500 cases). We used an original Python script written by A.S. as part of this review [50] for kappa calculation. The kappa calculation algorithm was reverse-engineered from Randolph’s original online application. If the lower margin of the kappa CI was higher than 0.6, we would consider piloting successful. Otherwise, we planned to discuss ways to improve agreement and conduct another piloting procedure in the same way as the initial one. The repeated piloting would be considered successful, regardless of its kappa value, to save resources. High inter-rater agreement was observed: A.S.-A.P., kappa 0.92 (95% CI, 0.89–0.96); A.S.-A.L., kappa (95% CI, 0.94–0.98); A.S.-K.V., kappa 0.98 (95% CI, 0.96–1.00).

For actual screening, an original Python script was developed by A.S. [50] and used to split the deduplicated record set, save the pilot subset, into subsets of 500 and randomize them to ensure the homogeneity of the batches. Screener masking was implemented using the Rayyan software (https://www.rayyan.ai) [41] Blind mode, which ensures that screening decisions are invisible to other screeners. We did not discuss screening decisions before the independent screening stage was completed by all screeners. Screening by title and abstract was conducted independently and in duplicate by four screeners (A.S., A.P., A.L., K.V.), divided into three pairs (A.S.-A.P., A.S.-A.L., A.S.-K.V.). Randomized subsets were uploaded into Rayyan and assigned to the pairs. We did not plan to conduct periodic inter-rater agreement checks; however, high inter-rater agreement was observed during pilot screening, and randomization ensured the consistent composition of record batches throughout the screening process.

After screening by title and abstract, full texts were retrieved by A.S. For any reports not written in English or Russian, an attempt would be made to translate them using either Google Translate document (or image) translation or DeepL Translate Files [51], whichever was the first to provide a comprehensible result. Four authors (A.S., A.P., A.L., K.V.), working in pairs, assessed the full texts for eligibility both independently and in duplicate. Discrepancies were resolved by consensus. Finally, P.Z. collated the included reports by study.

#### 2.4.3. Data Collection Process

Form building and a further data extraction process was conducted via Sysrev [52], both independently and in duplicate, by two authors (A.S., P.Z.). Due to the low number of included studies (*n* = 3), the pilot data extraction form had a low yield, and we decided to complete data extraction in one step. Of note, our data extraction form was developed and published as part of the a priori systematic review protocol, so the review team was familiar with it. Both extractors filled in the forms and consolidated the data via discussion. No changes were made to the data extraction form, which remained as pre-specified and prospectively published in the PROSPERO registry and the review data repository. The data in the manuscript align with the contents of the form. Additionally, A.S. extracted and P.Z. cross-checked data from the Kaplan–Meier (KM) curves from the included reports using ChartDetective [53]. All disagreements were resolved after data extraction. All data were published in two formats, as extracted and as prepared for analyses (see the Data Availability Statement).

### 2.5. Data Items

We based our list of variables for extraction on the comprehensive list used by Itchaki et al. (2013) [37], with modifications (K.L., A.M.). A full listing is available from the review repository (see the Data Availability Statement). If few data were available in the trial reports to analyze these outcomes, we would consider all types of reported outcomes.

### 2.6. Outcomes and Prioritization

We used the approach outlined by Itchaki et al. (2013) [37], with modifications (K.L., A.M.). The primary outcome was progression-free survival (PFS) as defined by Cheson et al. (2007) [54]: “disease progression or death as a result of any cause measured from entry onto study”. We decided to choose PFS as the primary outcome, based on its widespread use as an indicator of the effectiveness of therapy in oncology. We were also guided by the features of the natural history of FL, namely its long indolent course, which makes the assessment of the key outcome in oncology—overall survival (OS)—difficult. This approach has potential limitations due to the fact that PFS is a surrogate endpoint and is not associated with the hard clinical endpoint—OS for all tumors. Secondary outcomes included OS, defined as “death as a result of any cause measured from entry onto study”, the interval between the start of study and treatment, the duration of response (remission duration), the ratio of complete response to partial response (CR/PR), the percentage relapsed out of CR, treatment-related toxicity (cardiotoxicity, myelosuppression, infections, alopecia, and stomatitis), and other adverse events as defined in the individual study.

### 2.7. Risk of Bias in Individual Studies

We used RoB 2.0 [55] for outcome-level risk-of-bias assessments. We did not plan any subgroup analyses by risk of bias due to a known low number of the included studies. A risk-of-bias graph and summary visualizations were created using the R package robvis version 0.3.0 [56].

### 2.8. Data Synthesis

We planned to conduct quantitative synthesis if we deemed patients’ characteristics homogenous across the included studies. Our most important concern was pooling newly diagnosed lymphoma with relapsed or refractory lymphoma, which would not arguably comprise a clinically meaningful population (K.L.). Another important concern was the type of vaccine used: the pooling effects of autologous and allogeneic vaccines could be controversial (A.M.). If quantitative synthesis was not appropriate, we would descriptively summarize the contents of the data extraction form for each study and elaborate on these findings in the discussion. 

We based our quantitative analysis strategy on the report by Ossendorf et al. (2011), with modifications. We used I^2^ to measure heterogeneity, with I^2^ > 30% deemed moderate heterogeneity and I^2^ > 75% deemed considerable heterogeneity. We performed meta-analyses using a fixed-effects model because we did not expect unexplained heterogeneity given the similar populations, interventions, and design of the trials. However, given the heterogeneity that was observed, we also presented the results of meta-analyses carried out using the random-effects model, but we did not use them in the Summary of Findings table to adhere to the original systematic review protocol. We did not plan to conduct additional analyses due to the low number of included studies but did conduct several analyses out of necessity (see Section 3.9). All analyses were conducted in R by A.S. and checked by P.Z., notably using the packages survival version 3.5-7 [57,58], meta version 6.5-0 [59], survminer version 0.4.9 [60], and forestplot version 3.1.3 [61]; raw R code is available from the review repository (see the Data Availability Statement).

### 2.9. Meta-Bias(es)

We assessed the risk of publication bias using a contour-enhanced funnel plot, generated using the R package meta [59]. We assessed the risk of bias due to missing evidence using ROB-ME [62].

### 2.10. Confidence in Cumulative Evidence

We determined certainty of evidence using the GRADE guidance for systematic reviewers [29]. We summarized the review findings in a GRADE Summary of Findings table (v3), generated using the GRADEpro Guideline Development Tool [63].

## 3. Results

### 3.1. Summary of Findings

Three randomized controlled trials (RCT) met the inclusion criteria. The total number of patients was 813. The primary outcome was PFS. A meta-analysis showed that the hazards of disease progression in the vaccine group were higher than in the placebo group, but this difference was not statistically significant: Hazard ratio (HR) of 1.09 (95% CI, 0.91 to 1.3, *p* = 0.36). The certainty of evidence was moderate (Table 1).

### 3.2. Study Selection

**Figure 1:**
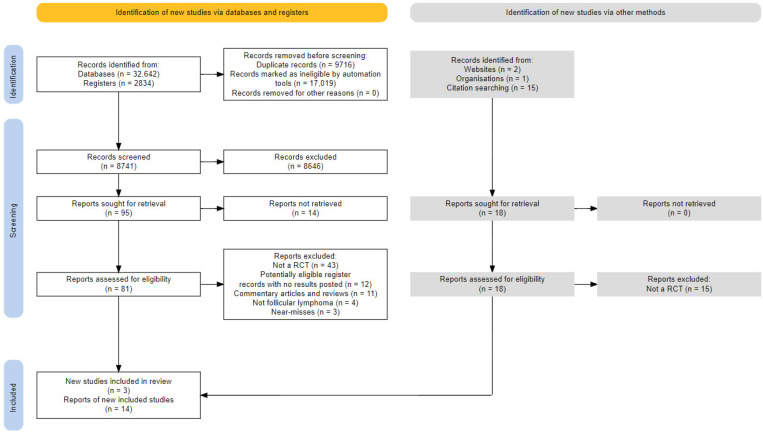
PRISMA 2020 flow diagram.

Searches were conducted on 17 December 2022. As reported in the flow diagram (Figure 1), we found 35,476 records in databases and registers (32,642 from databases, 2834 from registers; complete bibliographies for each information source are available from the review repository—see the Data Availability Statement).

After the removal of 9716 duplicates, 25,760 records remained for screening. After applying the RobotSearch classifier and manually correcting the output RIS file for end-of-record (ER) tags, which were corrupted for some records, 17,019 records were removed, and 8741 potential RCT records were selected and then screened manually.

After screening, 95 records were deemed eligible for inclusion, for which we were able to retrieve 81 full-text reports (a complete bibliography of the reports that we failed to retrieve is available from the review repository—see the Data Availability Statement). Two of the retrieved reports were in languages other than English: one in German [64] and one in Spanish [65]. Both were successfully machine-translated for the purpose of eligibility assessment. After the full-text screening, 11 reports (8 publications and 3 electronic supplemental materials), collated into 3 studies, were included: BiovaxID (BV) [34,56,57,58,59,60,61,62,63,64,65,66,67,68,69,70], Specifid (SP) [33] (also known as FavId), and MyVax (MV) [35]. After a review of the included reports for relevant references, additional Google and trial registry searches, a forward citation search, and manual searches, 3 more eligible associated reports were included (BV [71]; SP [72,73]), leading to a total of 14 reports. In particular, the forward citation search was unhelpful, and the manual review yielded one additional report [72], a preliminary report of the SP trial, which did not contribute additional information. A full report of the forward citation and manual searches is available from the review repository (see the Data Availability Statement).

A total of 73 reports identified via databases and registers, including both machine-translated reports, were deemed ineligible (a complete bibliography is available from the review repository—see the Data Availability Statement). The reasons for exclusion were as follows: not an RCT (n = 43); potentially eligible register records with no results posted (n = 12); commentary articles and reviews (n = 11); did not include patients with FL (n = 4); and 3 were “near-misses” (reports that appeared but were not eligible). During backward citation searches, we screened and excluded 15 more reports, all for one reason—“not an RCT”.

The following reports were among the near-misses: A report by Santos et al. (2005) [74] appeared to meet the eligibility criteria and was related to one of the three included studies (BV); however, this poster session actually reported on the outcome of a non-RCT phase II study with a brief mention of the phase III RCT, and the reported information was later superseded by more recent reports. A report by Timmerman et al. (2009) [75] was similar: it reported a phase I/II trial while mentioning a phase III trial (MV). Finally, a publication by Koc et al. (2004) [76] reported a non-controlled trial of the SP vaccine while noting that a phase III trial was underway.

We contacted the corresponding authors of the three included trials via email three times over a period of six weeks and asked them to share individual participant data. One author responded (SP) and advised that they did not have the primary data because the trial was sponsored by a pharmaceutical company (Favrille). Additionally, we filed a Freedom of Information Act (FOIA) request to the U.S. Food and Drug Administration (FDA) to retrieve Clinical Study Reports. In response, an FDA representative advised that the documentation related to these three trials is not disclosable under the FOIA because the drugs were not approved by the FDA.

### 3.3. Patient Characteristics

All three included studies were multicenter RCTs performed between 2004 and 2014, which enrolled a total of 870 participants. The patient groups were comparable in their characteristics (Table 2). However, MV and BV included only treatment-naïve patients, whereas SP participants were untreated or developed relapsed or refractory disease after prior chemotherapy.

### 3.4. Study Designs

In each study, participants received chemotherapy before vaccination, but the regimens differed between studies: BV used Prednisone, Doxorubicin, Cyclophosphamide, and Etoposide (PACE) (min 6 cycles) or Rituximab + Cyclophosphamide, Doxorubicin, Vincristine, and Prednisone (R-CHOP) regimens; SP used rituximab (4 doses weekly for 4 weeks); and MV participants underwent eight cycles of the Cyclophosphamide, Vincristine, and Prednisone (CVP) regimen. Chemotherapy in each study was followed by a rest period, after which those patients who achieved CR or CR unconfirmed (CRu) (or PR in SP and MV) were randomized into two arms: vaccine and control. Randomization in BV and MV was completed in a 2:1 ratio in favor of the vaccine group; in SP, the ratio was 1:1. The drugs were administered in courses, and their composition was similar in all studies. Differences were present in the number of courses and the time frame of therapy were. For example, BV participants underwent five courses (4 days once a month) over 6 months. In the SP trial, courses were repeated monthly for the first six doses, every 2 months for the second six doses, and every 3 months for the third six doses until disease progression or excessive toxicity. MV participants were given seven courses at monthly intervals. Follow-up examinations of participants were similar in all studies and included physician assessment, CT scans, and blood tests. A detailed comparison of the study designs is available in Table 3.

### 3.5. Outcomes

PFS as an outcome was only explicitly reported in MV. The definition of PFS in this study was: “time from random assignment to the earliest time point identifying progression or death resulting from any cause”. In the BV study, disease-free survival (DFS) was chosen as the primary endpoint and was described by the authors as “calculated from date of randomization until date of relapse or last follow-up”. For SP, the primary endpoint was time to progression (TTP), described by the authors as “measured from the date of random assignment to the date of first documentation of PD [progressive disease], initiation of another therapy for lymphoma, or death as a result of lymphoma”. These three definitions are slightly different but close in meaning; for this reason, we considered it appropriate to perform a meta-analysis.

The secondary outcomes described in the reports included the following: for BV, OS, safety, immunologic, and molecular responses; for SP, objective response rate (ORR), rate of response improvement (RRI) (defined as “the percentage of patients with SD or a PR after rituximab whose response subsequently improved to a PR or CR”), duration of response, and safety; for MV, subsequent antilymphoma-therapy-free survival (SALT-FS) (defined as “time from random assignment to SALT or death resulting from lymphoma”), humoral immune response (IR), and safety.

### 3.6. Risk of Bias in Studies

**Figure 2:**
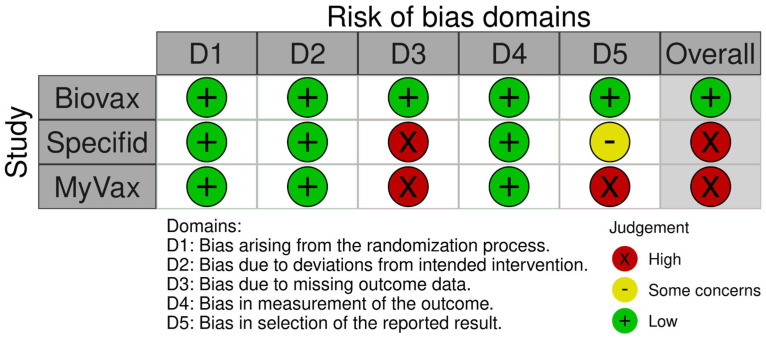
Risk of bias traffic lights graph.

**Figure 3:**
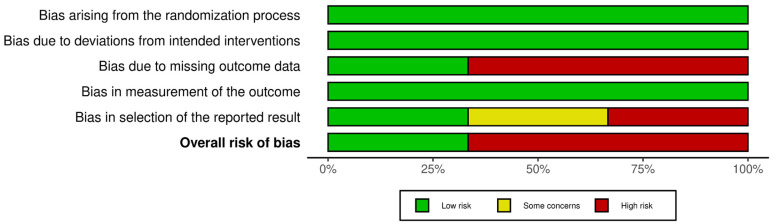
Risk of bias summary graph.

Risk-of-bias judgments are summarized in Figure 2 and Figure 3.

#### 3.6.1. Bias Due to Randomization

Allocation in all studies was centralized and randomized. The allocation sequence was also concealed until participants were enrolled and assigned to interventions. 

For two studies (BV, MV), baseline differences were not identified between intervention groups. In SP, there was a significant imbalance between groups for the FLIPI risk scores (Fisher exact test *p*-value = 0.0079), but the FLIPI components’ scores that were described in the article did not differ between groups. Thus, the difference in FLIPI scores was considered compatible with chance or flawed reporting but unlikely to have arisen from any randomization issues because, in this case, other baseline differences would have to be expected.

#### 3.6.2. Bias Due to Deviation from the Intended Interventions

In all three studies, participants, caregivers, and professionals delivering the interventions were not aware of participants’ assigned interventions during the trial. However, the SP report contained insufficient information on masking to draw an unequivocal conclusion. For the outcomes we assessed, the analyses were performed correctly in all three studies.

#### 3.6.3. Bias Due to Missing Outcome Data

In the BV study, data were available for 97% of participants, so the risk of bias in this case is low.

Regarding SP, we were uncertain of the number of participants for whom data were missing. For example, the study stated that “312 patients comprised the efficacy-assessable population”, which implies that data are missing for 37 participants out of 349 (11%). However, in a table in the report, a column labeled “permanently censored” included 49 participants (14% of the total). The outcomes for this group of participants were not reflected in the report; no explanation was provided in the report, so the data are not likely to be missing at random. Taking all of the above into account, the risk of bias in this case should be considered high.

The MV study did not report the number of participants who were available for PFS assessment, but such data were reported for IR assessment (*n* = 270), an outcome that was not considered in our review. Also, the MV study protocol contained an accurate description of the censoring process, but these data were not reported. Under the assumption that the number of participants available for PFS assessment was equal to the number available for IR assessment, data would be missing for 17 participants (6% of the total of 287), which is insignificant. However, this assumption is unfounded, so the risk of bias in this case should be considered high.

#### 3.6.4. Bias Due to Outcome Measurement

The method used to measure the outcome was appropriate and did not differ between intervention groups for all three studies. In all three studies, outcome assessors were not aware of the intervention received by study participants.

#### 3.6.5. Bias Due to Selection of the Reported Results

In the BV study, data were analyzed according to the study protocol, developed before the data became available for analysis. Also, the results were measured in the only way possible, and all eligible reported results for the outcome measurement corresponded to all intended analyses. Therefore, the risk of bias in this case is low.

For SP, no protocol was available, but the results were measured in the only way possible, and all eligible reported results for the outcome measurement corresponded to all intended analyses. Therefore, the risk of bias in this case can be assessed as “some concerns”.

In the MV study, the intended data analysis described in the protocol differed from the analysis given in the report. In addition, the authors of the study reported only one of the two planned PFS analysis methods. Taking these concerns into account, the risk of bias for the MV study was rated as high.

### 3.7. Results of Individual Studies

The effect estimation was conducted in several ways. First, hazard ratio (HR) and confidence interval (CI) data were extracted directly from the text of the reports. For BV, the difference between PFS in the two groups was not statistically significant, and the HR was 0.81 (95% CI, 0.56–1.16, *p* = 0.256). The same result was obtained for MV, where the HR was equal to 0.98 (95% CI, 0.72–1.33, *p* = 0.89). In SP, PFS was statistically significantly higher at the significance level α = 0.05 in the placebo group, compared to the vaccine group, HR = 1.384 (95% CI, 1.053–1.819, *p* = 0.019); however, the authors used a significance level α = 0.01. Alternative effect estimates derived from the individual studies and used in additional syntheses are reported below.

### 3.8. Results of Syntheses

**Figure 4:**
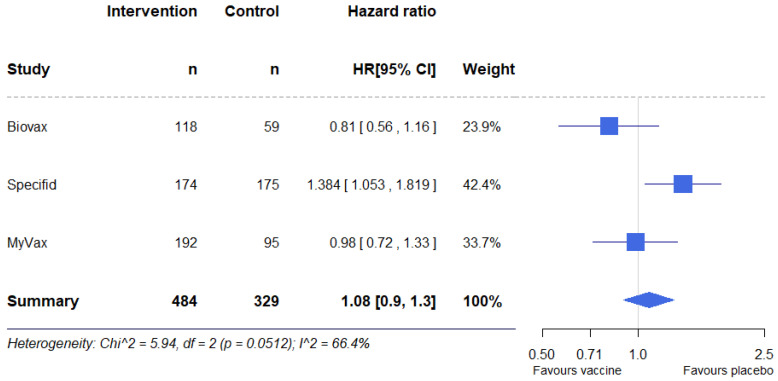
Forest plot for the meta-analysis of reported hazard ratios.

The results of individual studies were pooled into one meta-analysis, with a total of 813 participants (control group 329, vaccine group 484) (Figure 4). A meta-analysis performed using the fixed-effects model showed no statistically significant differences in PFS between the groups, HR = 1.08 (95% CI, 0.9–1.3, *p* = 0.37). There was significant heterogeneity between studies; I^2^ was 66.4%. A meta-analysis performed using the random-effects model also showed no statistically significant differences in PFS between the groups, HR = 1.05 (95% CI, 0.77–1.43, *p* = 0.76).

However, this meta-analysis cannot be considered conclusive because different methods of HR calculation were used in the studies. In BV, HR was obtained using the logrank test. SP used a Cox regression model adjusted for prior therapy and disease response to rituximab. Finally, MV reported that both the logrank and Cox regression model were used, adjusted for demographic and baseline characteristics. 

**Figure 5:**
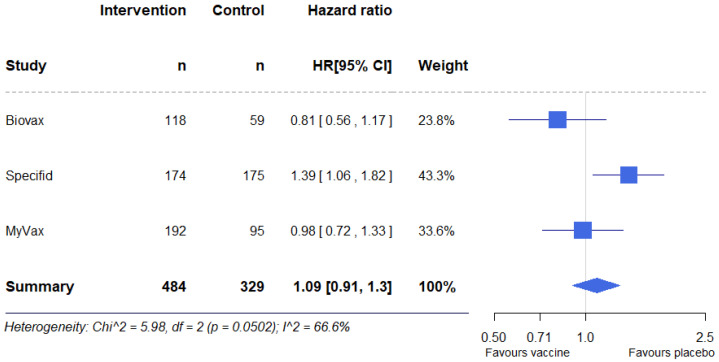
Forest plot for the meta-analysis of recalculated effect estimates.

In light of these considerations, we conducted another meta-analysis using effect estimates recalculated from other data from the reports: *p*-value, number of total events, number of participants in control arm, and number of participants in vaccine arm [77]. With these data, we obtained similar HRs for all three studies: BV, 0.81 (95% CI, 0.56–1.17, *p* = 0.256); SP, 1.39 (95% CI, 1.06–1.82, *p* = 0.019); MV, 0.98 (95% CI, 0.72–1.33, *p* = 0.89). A fixed-effects model meta-analysis also showed no statistically significant differences in PFS between the groups: HR = 1.09 (95% CI, 0.91–1.30, *p* = 0.36) (Figure 5). Heterogeneity between studies was also high; I^2^ = 66.6%. In this case, using the random-effects model also did not reveal statistically significant differences in PFS between groups, HR = 1.05 (95% CI, 0.77–1.44, *p* = 0.76).

### 3.9. Additional Analyses

**Figure 6:**
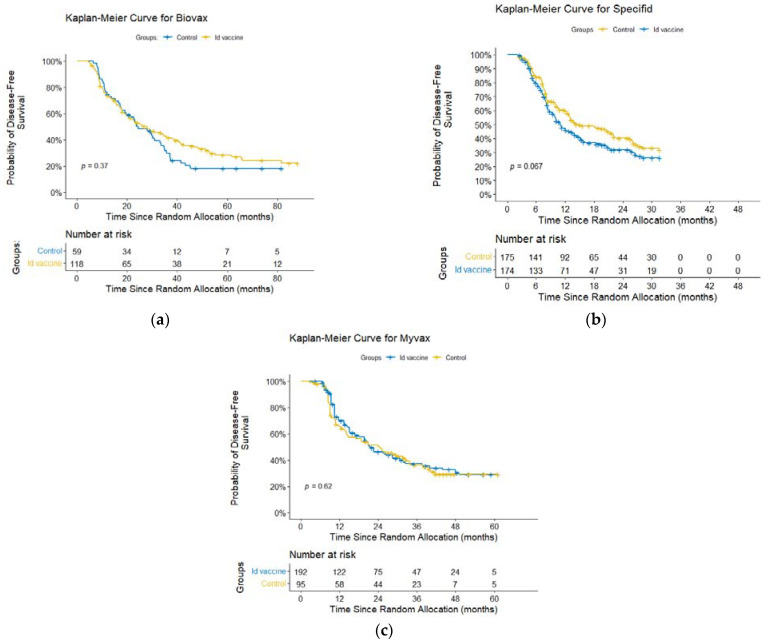
Reconstructed Kaplan–Meier curves: (**a**) BiovaxID; (**b**) Specifid; (**c**) MyVax.

We also performed an additional meta-analysis of PFS in a third way, as suggested by the Cochrane Handbook [29]. We reconstructed individual participant data (IPD) from KM curves and additional data from reports [78]. We obtained reconstructed KM curves and HR estimates using a Cox regression model without adjustments. Visually, the KM curves appeared to be recovered fairly accurately for all the three studies. No statistically significant differences in PFS between the groups were found in any study: BV, HR = 0.85 (95% CI, 0.59–1.21, *p* = 0.37) (Figure 6a); SP, HR = 1.28 (95% CI, 0.98–1.68, *p* = 0.067) (Figure 6b); MV, HR = 1.07 (95% CI, 0.78–1.46, *p* = 0.62) (Figure 6c).

**Figure 7:**
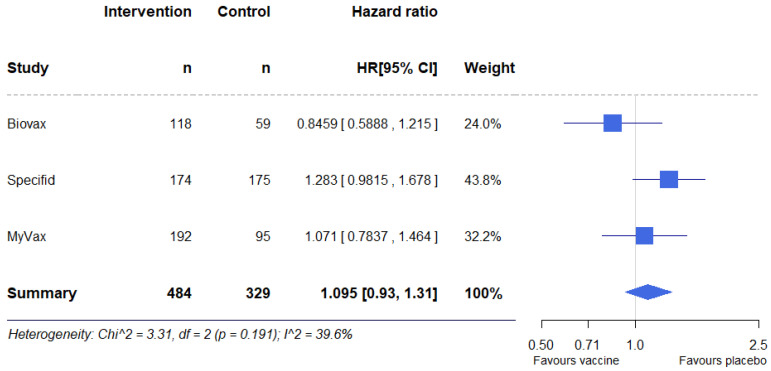
Forest plot for the meta-analysis of reconstructed individual participant data.

A fixed-effects model meta-analysis also showed no statistically significant differences in PFS between the vaccine and control groups; HR = 1.095 (95% CI, 0.93–1.31, *p* = 0.32) (Figure 7). Heterogeneity was low; I^2^= 39.6%. The results of a meta-analysis performed using the random-effects model were also not statistically significant, HR = 1.07 (95% CI 0.86–1.36, *p* = 0.52).

**Figure 8:**
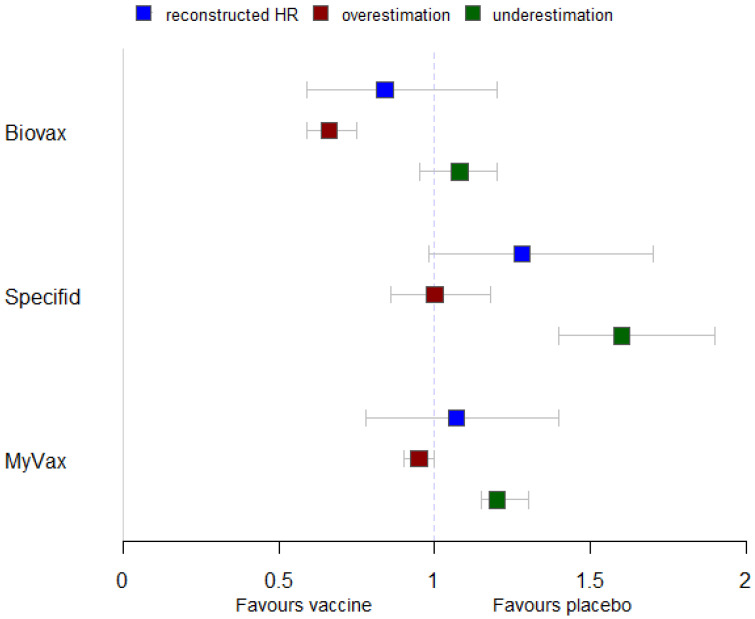
Sensitivity analyses of hazard ratios reconstructed from Kaplan–Meier curves. overestimation, true hazard ratios (HRs) in the overestimation case; underestimation, true HRs in the underestimation case. Whiskers represent 95% confidence intervals (95% CIs).

Based on a previously published HR reconstruction methodology [77], a degree of imprecision was expected in the reconstructed HR and CI estimates. Therefore, we performed a sensitivity analysis. We investigated an extreme case of underestimation (i.e., with the true value being equal to HR + mean absolute HR reconstruction error), as well as an extreme case of overestimation (i.e., with the true value being equal to HR—mean absolute HR reconstruction error) of the reconstructed hazards, with narrow CIs (standard error (SE)—mean absolute error of SE) (Figure 8).

In the underestimation case, PFS was statistically significantly greater in the placebo group; pooled HR = 1.28 (95% CI, 1.01–1.60, *p* = 0.04) (not shown in the figure). In the overestimation case, no statistically significant difference in PFS between the two groups was observed; pooled HR = 0.86 (95% CI 0.67–1.10, *p* = 0.24) (not shown in the figure). Heterogeneity was high in both cases: I^2^ was equal to 87.7% and 92.8%, respectively.

In summary, the reconstructed IPD was not susceptible to overestimation but could have been underestimated, leading, in an extreme case, to a missed significant pooled hazard ratio favoring the placebo group.

### 3.10. Safety

In terms of safety, all three studies reported that vaccination was well-tolerated by participants. Among the most common adverse events for all three studies were the following: injection site reactions, fatigue, fevers, chills, nausea, headache, muscle pain, and arthralgia.

The SP study did not offer a categorization of side effects by grade but did report that the type, frequency, and severity of side effects were comparable between arms.

In the BV study, for grade 1–2 adverse events that occurred in ≥10% of participants, differences in frequency between the arms were observed only for infection (21.1% in the vaccine group versus 4.9% in the placebo group, *p* = 0.029, two-sided Fisher exact test). In terms of grade 3–4 adverse events (17 cases for the vaccine arm, 13 cases for the placebo arm), there was no statistically significant difference between the groups for the overall rate of adverse events (*p* = 0.331, two-sided Fisher exact test).

The MV study reported only the number of adverse events by maximum grade, without specifying the adverse events, except for the most frequent ones. Overall, the distribution of adverse events was similar in both groups. For grade 1–2 adverse events, the distribution was as follows: 88.6% of the total number of adverse events in the vaccine group versus 87.3% in the placebo group; as to grade 3–4 adverse events, 10.9% vs. 10.7% respectively.

None of the studies reported drug-related deaths.

### 3.11. Reporting Biases

The ROB-ME tool [62] was used to assess the risk of bias due to missing evidence. The results of each study that met our inclusion criteria were available for inclusion in the meta-analyses. All studies were identified and deemed eligible for inclusion in the meta-analyses before the results of the studies were generated or became known. We found all studies met the inclusion criteria for this systematic review, regardless of their results. Therefore, the risk of bias due to missing results can be assessed as low.

**Figure 9:**
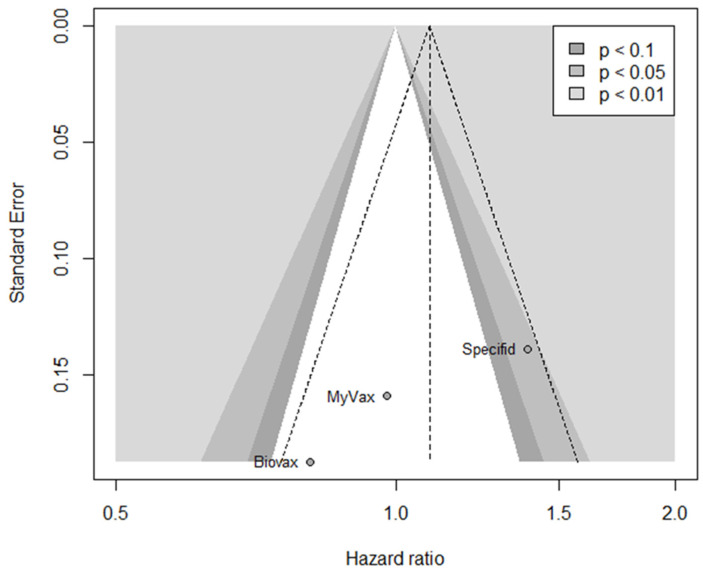
Contour-enhanced funnel plot.

Small-study effects were assessed by using a contour-enhanced funnel plot (Figure 9). The small number of studies complicated its interpretation; however, publication bias seems to shift the estimate in favor of the control group. This finding is not surprising given that the largest and only study that showed a statistically significant result, SP, favored the control group.

### 3.12. Certainty of Evidence

Two of the three RCTs had a high risk of bias due to missing outcome data: the SP study had missing outcome data for 14% of participants, and the MV study did not explicitly state the number of participants for whom the outcome was estimated. Also, for SP, the study protocol could not be found, making an assessment of bias due to the selective reporting of outcomes impossible. In the MV study, the authors deviated from the analysis plan described in their study protocol, which makes the risk of bias high. Given this, the certainty of evidence could be downgraded by one level.

The results of the studies have considerable heterogeneity. This may be explained by differences in the groups of participants (treated and untreated) and differences in the number of courses of therapy. The presence of such a significant heterogeneity may also be explained by a low number of included studies. In addition, the BV study only randomized patients who achieved a CR after prevaccination therapy, whereas the other two studies also randomized those who achieved only a PR. In the BV study, idiotype (Id) protein was produced using a hybridoma, whereas in SP and MV, recombinant Id was used. Prevaccination regimens also varied, with MV using the CVP regimen, SP using rituximab alone, and BV using either the PACE or CHOP regimen. Overall, we did not feel that much unexplained heterogeneity was present and, therefore, did not downgrade the certainty of evidence for inconsistency.

We did not identify important issues with the indirectness of evidence, imprecision, and publication bias, so we did not downgrade the certainty of evidence based on any of these.

Thus, the overall certainty of the evidence can be rated as moderate.

## 4. Discussion

This systematic review was conducted to evaluate the efficacy and safety of therapeutic vaccines against FL. We reviewed RCTs investigating vaccines for FL of any therapeutic design. Three RCTs involving 813 participants were found to fit the inclusion criteria. All three studies focused on the same type of vaccine, the Id vaccine. We were able to show that Id vaccines for FL do not differ in efficacy from control therapy. Moreover, the vaccination was well tolerated by the patients, as indicated by the lack of difference between the vaccine and control groups.

The main limitation of this review is the small number of studies, all of which focused on Id vaccines. The included studies were characterized by bias due to missing outcome data, differences in patient groups (treated and untreated), and differences in the number of vaccination courses. In addition, in the BV study, only patients who achieved a CR after prevaccine therapy were randomized, whereas in the other two studies, those with a PR were also randomized. In the BV study, the Id protein was produced using hybridoma, whereas in SP and MV, recombinant Id was used. Prevaccine therapy regimens also differed, with MV using the CVP regimen, SP using rituximab alone, and BV using either the PACE or CHOP regimen. Also, all three studies used different primary endpoints: DFS was used in BV, TTP in SP, and MV in PFS. Although the authors provided definitions for these, and they are similar, and the described ways of assessing the endpoints also appear to be similar, such differences still may pose problems when interpreting the results. The authors provide only a limited description of the side effects observed in their studies, and the differences in these descriptions make generalization and comparison difficult.

There are also limitations associated with the review processes used. We searched an extensive list of bibliographic databases and clinical trial registers, including several non-English language databases such as the KCI-Korean Journal Database and SciELO Citation Index; however, many databases in other languages exist, such as databases in Chinese, which we were unable to search due to language and cross-border access barriers. We were unable to obtain the full texts of all the articles retained after screening, which may have included suitable studies. Also, we were unable to obtain primary data; we made attempts to contact the study authors, but they either did not respond or were unable to provide the data. In addition, for the SP study, we were unable to find the study protocol, which made it difficult to assess the risk of bias. However, none of these methodological difficulties is likely to have significantly affected the overall conclusion of our review.

Although our intention in initiating this work was to evaluate the efficacy of all existing FL vaccine types, and we made every effort to find all suitable RCTs, our results can only be applied to Id vaccines. Given that we carefully followed the up-to-date guidelines for performing systematic reviews, we can assume that RCTs focusing on other vaccine designs are not available at the time of writing the review. Disappointingly, Id vaccines, despite their initial promise, were found to be ineffective for FL therapy. Thus, two of our expectations were not met: First, the set of FL vaccine types that have reached the phase III trial stage is limited to Id vaccines. Second, despite the apparent promise of this approach, the data suggest that it is ineffective. The lack of prospects of vaccination, in its current form, for FL therapy is confirmed by the withdrawal of all three companies from the market after the unsuccessful completion of trials of their drugs. Another argument is the small number of related studies: we managed to find only one study [79] devoted to this type of vaccine, which was conducted after 2014 (the year of publication of the last RCT).

Although Id vaccines have not been shown to be effective against FL, other therapeutic vaccine types are still a focus of research. Thus, the idea of Id vaccines has evolved in the form of an attempt to inject DNA with an encoded Id [80,81]. The peptide vaccine technology is also being actively studied. For example, Uffe Klausen et al. (2021) used checkpoint molecules PD-L1 and PD-L2 for immunization [82]. Cell-based vaccines have been the topic of research for almost as long as Id vaccines, but none of them have yet reached the stage of phase III studies. Nevertheless, there are several studies focusing on this technology at various stages at present (NCT03035331, NCT01239875, NCT00487305, NCT02194751, NCT02677155).

A new approach to creating therapeutic antitumor vaccines, namely, the use of neoantigens for the induction of immune response, is also being actively investigated for FL therapy. Neoantigens are products of mutated tumor genes, are specific for tumor cells, and are not expressed on the surface of healthy cells, so the limitations of tumor-associated antigens in the form of the development of immune tolerance are not typical. At present, there are two studies on this type of vaccine: NeoVax and EO2463 (NCT03361852, NCT04669171). NeoVax, moreover, has been shown to be effective for patients with melanoma [83] and glioblastoma [84].

## 5. Conclusions

In conclusion, we discovered that all RCTs of therapeutic vaccines against FL focused on one technology—Id vaccines. Moreover, Id vaccines have not been shown to be effective as an FL therapy. However, vaccination, as an approach, may still be promising for FL. The efficacy of antitumor vaccination can potentially be improved by using personalized neoantigens as a method of inducing an immune response and by using novel antigen delivery vehicles, e.g., DNA or RNA.

## Figures and Tables

**Table 1:** Summary of findings table. Patient-specific recombinant idiotype keyhole limpet hemocyanin (Id-KLH) vaccine compared to KLH-KLH placebo for follicular lymphoma, treated or untreated. Patient or population: follicular lymphoma, treated or untreated. Setting: clinical trial, post-chemotherapy treatment combined with granulocyte–macrophage colony-stimulating factor (GM-CSF). Intervention: patient-specific recombinant idiotype keyhole limpet hemocyanin (Id-KLH) vaccine. Comparison: KLH-KLH placebo.

Outcome N° of Participants (Studies)	Relative Effect (95% CI)	Anticipated Absolute Effects (95% CI)			Certainty	What Happens
		With Placebo	With Vaccine	Difference		
Progression-free survival (PFS) assessed with: Hazard Ratio follow-up: median 42.8 months N° of participants: 813 (3 RCTs)	HR 1.09 (0.91 to 1.3) [Progression-free survival]	With baseline risk from the BiovaxID trial			⨁⨁⨁◯ Moderate	Therapeutic antitumor vaccines likely result in little to no difference in progression-free survival, with a non-significant increase in hazards of disease progression with vaccines.
		78.0%	80.8% (74.8 to 86)	2.8% more (3.2 fewer to 8 more)		
		With baseline risk from the Specifid trial				
		57.0%	60.1% (53.6 to 66.6)	3.1% more (3.4 fewer to 9.6 more)		
		With baseline risk from the MyVax trial				
		63.0%	66.2% (59.5 to 72.5)	3.2% more (3.5 fewer to 9.5 more)		

The risk in the intervention group (and its 95% confidence interval) is based on the assumed risk in the comparison group and the relative effect of the intervention (and its 95% CI). CI: confidence interval; HR: hazard ratio. GRADE Working Group grades of evidence. High certainty: we are very confident that the true effect lies close to that of the estimate of the effect. Moderate certainty: we are moderately confident in the effect estimate: the true effect is likely to be close to the estimate of the effect, but there is a possibility that it is substantially different. Low certainty: our confidence in the effect estimate is limited: the true effect may be substantially different from the estimate of the effect. Very low certainty: we have very little confidence in the effect estimate: the true effect is likely to be substantially different from the estimate of effect.

**Table 2:** Patient characteristics.

All Patients	BiovaxID	Specifid	MyVax
Newly diagnosed, relapsed, or refractory disease?	All treatment-naïve	Treatment-naïve and relapsed refractory	All treatment-naïve
Number of patients randomized	177	349	287
Age	Mean, 49.5 SD, 10.4	Median, 54 Range, 21–86	Median, 50 Range, 23–80
Female, %	45.7	43.3	54.7
Previous treatment	Previously untreated (except: prednisone for < 2 months, radiation alone)	No more than two systemic lymphoma therapies	Previously untreated
FLIPI (risk group, %)	Low or intermediate, 87.6 High intermediate or high, 12.0 Unknown, 0.4	Low, 37.0 Intermediate, 39.3 High, 22.3 Unknown, 1.4	Low, 10.0 Intermediate, 53.3 High, 36.0 Unknown, 0.7
Grade, %	Grade 1, 45.7 Grade 2, 53.4 Unknown, 0.9	Grade 1, 52.0 Grade 2, 41.0 Grade 3 (or unknown), 7.0	Grade 1, 51.0 Grade 2, 39.0 Grade 3, 7.0 Unknown, 3.0
Stage, %	Stage 2, 2.9 Stage 3, 26.5 Stage 4, 69.7	Stage 1, 2.0 Stage 2, 11.0 Stage 3, 40.1 Stage 4, 46.0 Unknown, 0.9	Stage 3, 40.4 Stage 4, 59.6
Histologic confirmation	All	All	All
ECOG performance status, %	ECOG 0, 75.6 ECOG 1, 23.1 ECOG 2, 0.9 Unknown, 0.4	ECOG 0, 85.4 ECOG 1, 14.3 ECOG 2 (or unknown), 0.3	ECOG 0, 70.0 ECOG 1, 28.6 Unknown, 1.4
All patients	BiovaxID	Specifid	MyVax

ECOG, Eastern Cooperative Oncology Group; FLIPI, Follicular Lymphoma International Prognostic Index; SD, standard deviation.

**Table 3:** Study designs.

Study Characteristic	BiovaxID	Specifid	MyVax
Pre-vaccination therapy	PACE (min 6 cycles) or R-CHOP	Rituximab mg/m^2^ weekly for 4 weeks	CVP 8 cycles
Response status after pre-vaccination therapy	CR or unconfirmed CR	SD, PR, or CR	PR, CR, or unconfirmed CR
Randomization parameters	Randomization 2:1 in favor of the vaccine group Patients were stratified by IPI group (0–2 vs. 3–4) and number of chemotherapy cycles given (≤8 vs. >8)	Randomization 1:1 Patients were stratified by prior treatment (treatment-naïve vs. relapsed/refractory disease) and response to rituximab therapy (CR/PR vs. SD)	Randomization 2:1 in favor of the vaccine group Patients were stratified by study site and disease response status (CRu/PR vs. CR)
Vaccine manufacturing details	Isotype-matched (IgM/IgG) Id protein manufactured using a heterohybridoma technology	Isotype-matched Id protein manufactured using a proprietary recombinant technology	Isotype-matched Id protein manufactured using a recombinant technology
Therapy by study arm	Vaccine: 0.5 mg ID-KLH (1 day) + GM-CSF 100 mcg/m^2^/d (1 to 4 days) Control: 0.5 mg KLH + GM-CSF 100 mcg/m^2^/d (1 to 4 days)	Vaccine: 0.5 mg ID + 0.5 mg KLH (1 day) + 250 mcg/d GM-CSF (1 to 4 days) Control: 0.5 mg KLH + 250 mcg/d GM-CSF (1 to 4 days)	Vaccine: 0.5 mg ID + 0.5 mg KLH (1 day) 250 mcg/d GM-CSF (1 to 4 days) Control: 0.5 mg KLH + 250 mcg/d GM-CSF (1 to 4 days);
Therapy regimen	Five ID vaccine or control injections at 1, 2, 3, 4, and 6 months	Monthly for first 6 doses, every 2 months for next 6 doses, and then every 3 months until evidence of progressive disease or unacceptable toxicity was observed	Seven ID vaccine or control injections at 4-week intervals over a period of 24 weeks
Primary outcomes	DFS	TTP	PFS
Secondary outcomes	OS, safety, immunologic, and molecular responses	ORR, RRI, duration of response, safety	SALT-FS, IRS, safety
Study characteristic	BiovaxID	Specifid	MyVax

CR, complete response; CVP, Cyclophosphamide, Vincristine, and Prednisone; DFS, disease-free survival; GM-CSF, granulocyte-macrophage colony-stimulating factor; ID, idiotype; IPI, International Prognostic Index; IRS, immune response; KLH, keyhole limpet hemocyanin; ORR, objective response rate; OS, overall survival; PACE, Prednisone, Doxorubicin, Cyclophosphamide, and Etoposide; PFS, progression-free survival; PR, partial response; R-CHOP, Rituximab + Cyclophosphamide, Doxorubicin, Vincristine, and Prednisone; RRI, rate of response improvement; SALT-FS, subsequent antilymphoma-therapy-free survival; SD, stable disease; TTP, time to progression.

## Data Availability

Raw search, screening, and data extraction data, as well as risk of bias assessments and R code used for analyses, are available from the systematic review repository on Open Science Framework: https://doi.org/10.17605/osf.io/kbzfw (accessed on 15 February 2024).
